# Health-related quality of life following a clinical weight loss intervention among overweight and obese adults: intervention and 24 month follow-up effects

**DOI:** 10.1186/1477-7525-4-43

**Published:** 2006-07-17

**Authors:** Bryan Blissmer, Deborah Riebe, Gabriela Dye, Laurie Ruggiero, Geoffrey Greene, Marjorie Caldwell

**Affiliations:** 1 University of Rhode Island, Kingston RI 02881, USA; 2 University of Illinois, Chicago, USA

## Abstract

**Background:**

Despite a growing literature on the efficacy of behavioral weight loss interventions, we still know relatively little about the long terms effects they have on HRQL. Therefore, we conducted a study to investigate the immediate post-intervention (6 months) and long-term (12 and 24 months) effects of clinically based weight management programs on HRQL.

**Methods:**

We conducted a randomized clinical trial in which all participants completed a 6 month clinical weight loss program and were randomized into two 6-month extended care groups. Participants then returned at 12 and 24 months for follow-up assessments. A total of 144 individuals (78% women, M age = 50.2 (9.2) yrs, M BMI = 32.5 (3.8) kg/m^2^) completed the 6 month intervention and 104 returned at 24 months. Primary outcomes of weight and HRQL using the SF-36 were analyzed using multivariate repeated measures analyses.

**Results:**

There was complete data on 91 participants through the 24 months of the study. At baseline the participants scored lower than U.S. age-specific population norms for bodily pain, vitality, and mental health. At the completion of the 6 month clinical intervention there were increases in the physical and mental composite measures as well as physical functioning, general health, vitality, and mental health subscales of the SF-36. Despite some weight regain, the improvements in the mental composite scale as well as the physical functioning, vitality, and mental health subscales were maintained at 24 months. There were no significant main effects or interactions by extended care treatment group or weight loss group (whether or not they maintained 5% loss at 24 months).

**Conclusion:**

A clinical weight management program focused on behavior change was successful in improving several factors of HRQL at the completion of the program and many of those improvements were maintained at 24 months. Maintaining a significant weight loss (> 5%) was not necessary to have and maintain improvements in HRQL.

## Background

The number of Americans who are seriously overweight has reached epidemic proportions and is still on a rise [1]. Currently, 66.3% of the Americans are overweight and 32.2% are classified as obese [2]. Obesity is a complex disease resulting from the interaction of multiple factors: genetic, metabolic, social, behavioral, and cultural [3], and as such has dramatic effects on overall health and well-being of overweight or obese individuals.

Physically, some of the problems associated with obesity are hypertension, coronary arteriosclerosis, elevated cholesterol, type 2 diabetes, joint problems, stroke, and certain types of cancers [4,5]. Psychologically, obesity is associated with a myriad of problems including lower self-concept, negative self evaluation, and decreased self-image [6]. Socially, obese individuals often encounter discrimination and prejudice, which further perpetuate negative economic and social consequences [5]. In general, obesity is associated with decrements in overall quality of life whether it is physical, psychological, or social.

The impact of being overweight and obese has been studied from the perspective of health-related quality of life (HRQL). Although there is no standard definition of HRQL, it is generally accepted that it is a subjective, multidimensional assessment of the physical, psychological, and social domains of health [7]. There is a growing body of cross-sectional data that support a strong relationship between obesity and the quality of life, in that the quality of life seems to decrease as a function of weight increase [8-11].

In general, the literature has supported that even a small weight reduction often leads to significant improvements in HRQL [12]. Results of a recent meta-analysis on the effects of randomized controlled trials of weight loss on HRQL using a variety of intervention methods (behavioral, surgical, pharmacologic) suggests that the most consistent effects are found only when using obesity-specific measures of HRQL [13]. Our concern is that the majority of the population is in the overweight or moderately obese categories that may not really experience much limitation on an obesity-specific measure of HRQL. We know little of the HRQL effects programs might have on that more "typical" population, which is likely to start with better overall functioning and higher baseline levels of HRQL. In addition, the majority of the studies on HRQL changes in obese and overweight individuals have focused on major medical techniques, such as gastric bypass surgery, or pharmacotherapy [8]. Although these may be important strategies and options for severely obese individuals (Class III), the majority of the population is more likely to attempt a behavioral program focused on changing their dietary and exercise behaviors.

There have been relatively few studies that have examined the effects of lifestyle modification programs on changes in quality life among overweight and obese individuals. These studies (e.g., [11,14-16]) suggest that physical activity in combination with diet can be effective in improving health related quality of life in several domains including social functioning, mood, and self esteem. In general these studies note that obesity seems to have a greater impact on physical rather than mental functioning [12].

Therefore, current studies provide some evidence that a short term weight loss has a positive effect on health related quality of life; however, individuals who initially lose weight tend to regain much of the weight following the termination of the intervention [3]. More attention needs to be paid to long term weight loss maintenance because weight relapse prevention is crucial, and only a limited number of studies focus on the effects of weight loss maintenance on HRQL.

In one of the only studies to include a long-term follow-up of a lifestyle weight management program, Kaukua et al. [15] studied the effects of weight loss on HRQL longitudinally. Weight loss was achieved by placing obese and overweight individuals on very low energy diets. Participants attended a 4-month weight loss program, by the end of which they experienced weight loss and marked improvements on anthropometric measures as well as on most facets of HRQL. At the end of two years, most study participants regained weight, with 1/3 maintaining a weight loss of 5% of initial body weight [15]. Interestingly, the physical functioning subscale was the only HRQL subscale that remained improved at the 2 year follow-up. A separate measure of obesity related psychosocial problems also remained improved at the 2 year follow-up.

Kaukua et al. [15] also examined dose response effects by percentage weight loss maintained in their study participants at 2 years. They found evidence of a dose-response effect, with study participants that maintained a greater than 10% weight loss maintaining improvements in with both physical and mental subscales.

These findings suggest that only modest improvements in HRQL are observed with longer follow ups. However, these studies [14,15] utilized a very-low-energy diet approach designed to produce very rapid changes in weight among severely obese patients (mean BMI = 42.8). There is a need to investigate less severe caloric restriction approaches that incorporate healthy eating, exercise, and behavioral counseling among adults that are not severely overweight as this may be more typical of how we might treat the majority of overweight and obese adults.

Fontaine et al. [16] examined a 1-year follow-up to a weight loss program among 32 mildly to moderately obese adults. They found that increases immediately post intervention on many of the SF-36 subscales, but only general health and vitality remained improved at 1 year. In addition they found no difference in changes in HRQL between weight regainers and maintainers. These are interesting findings among more moderately obese adults, but need replication with a greater sample size to detect meaningful differences as well as longer periods of follow-up.

The focus of the current study was to investigate the immediate post-intervention (6 months) and long-term (12 and 24 months) effects of clinically based weight management programs on HRQL in overweight and moderately obese adults. Changes in HRQL were a secondary outcome of interest in a study designed primarily to investigate the efficacy of differential maintenance interventions on weight loss maintenance [17].

## Methods

### Participants

Men and women over the age of 18 with a BMI between 27–40 kg/m^2 ^volunteered to participate in this study. Prior to study enrollment, participants received written clearance from their primary care physician and provided written informed consent according to the Institutional Review Board at the University of Rhode Island. Participants completed a medical history questionnaire, binge eating questionnaire and the Beck Depression Inventory. Participants were excluded if exercise or dietary fat reduction was contraindicated for medical reasons, if they had active cancer or type 1 diabetes, or if they reported symptoms of an eating disorder or depression. In addition, participants underwent a symptom-limited exercise treadmill test to rule out the presence of significant cardiovascular disease.

### Clinical program

All participants completed a six month clinical weight management program. The multidisciplinary program, delivered to groups of 11–15 participants, focused on changing lifestyle rather than weight loss per se. The program began with an intensive three month phase during which participants attended two, two-hour sessions each week. Each session involved one hour of behavioral or dietary instruction and one hour of exercise. Following the intensive phase, participants attended a tapering phase where participants met for a total of eight one-hour visits over three months.

Details of the weight management program have been reported elsewhere [17]. Briefly, the program highlighted three key components: exercise, nutrition education and behavioral counseling. Supervised exercise sessions involved aerobic exercise conducted at 60–70% of measured maximal heart rate. Duration of the sessions gradually increased from 15 minutes to 45 minutes during the first 12 weeks of the program. Participants were instructed to exercise an additional two times per week outside of the program. The dietary intervention focused on healthy eating rather than dietary restriction. Participants were encouraged to set daily fat gram goals at 20, 25, or 30% of calories, monitor fat intake, increase their consumption of fruits, vegetables, and whole grains, and to follow the principles of balance, variety, and moderation. The behavioral component of the intervention was based on the principles and processes of the Transtheoretical Model [TTM; [18]]. Motivational and behavioral principles to modify eating patterns, to initiate and/or continue moderate exercise and to increase the activities of daily living were introduced. Stage-specific strategies were presented in a progressive fashion.

During the clinical program, participants received 3 computer-generated individualized expert system reports on TTM mediator variables at baseline, 3 and 6 months. The first two reports were distributed in the groups and reports were discussed as part of the groups process, the third report was delivered via mail. Participants also received reports about anthropometric, biochemical and dietary variables at baseline, 3, and 6 months.

### Extended care intervention

Prior to participation in the clinical program, participants were randomly assigned into one of two extended care intervention groups. Both groups attended the same 6-month clinical program and received identical reports about anthropometric, biochemical and dietary variables at 12 and 24 months. The extended care treatment group received two additional computer-generated, individualized TTM reports, via mail, at 9 and 12 months. The extended care comparison group received generic, action-oriented information about diet and exercise at the same two time points. There was no additional contact with participants during the 18-month follow period.

### Measures

All measures were collected at baseline, 6 months (end of clinical program), 12 months, and 24 months.

#### Anthropometrics

Body weight was measured on a calibrated electronic floor scale, and height was measured to the nearest 0.5 cm using a stadiometer. Skinfold thickness of the biceps, triceps subscapula, chest, abdomen and thigh were measured. Body density was calculated using the equations of Jackson and Pollock [19]. The percentage of body fat was estimated using the Siri [20] formula.

#### Health-Related Quality of Life

HRQL was assessed using the Medical Outcome Study (MOS) Short Form-36 (SF-36). The SF-36 contains eight scales (Physical Functioning, Role-Physical, Bodily Pain, General Health, Vitality, Social Functioning, Role-Emotional, and Mental Health) that are organized in a hierarchical manner to the summary measures of Physical and Mental Health. The highest possible scores on the eight subscales are 100, representing perfect functioning, and the summary scales have a t-score distribution. Each of the eight scales has been found to possess adequate reliability and validity across a number of studies and populations [21].

### Statistical analyses

One sample t-tests were used to examine baseline differences between the SF-36 scales and population norms. Repeated measures MANOVAs were used to examine the Time main effects for the SF-36 subscales as well as the composite scores. To examine potential effects of differential extended care group assignment, it was included as a between subjects factor. It should be noted that there were no differential effects on the primary weight outcomes by treatment assignment [17]. Based upon work in previous studies [e.g., [15]], study participants were also categorized into weight loss groups to compare those individuals that had, at 24 months, maintained at least a 5% weight loss from baseline (30%) versus those that had not (70%). Time points in the analyses included baseline, post intervention (6 months), 1 year, and 2 year follow-up.

## Results

### Subject characteristics

Table 1 shows the subject characteristics for individuals who completed the 6-month clinical program. The study population consisted mostly of educated, Caucasian (97%) men and women. Seventy-eight percent of the study participants were female. All participants were considered overweight or obese with a BMI above 27 kg/m^2^.

**Table 1:** Descriptive characteristics of participants that completed the 6-month clinical program (n = 144)

Age (yr)	50.2 (9.2)
Height (cm)	165.8 (9.0)
Weight (kg)	89.7 (14.9)
BMI (kg/m^2^)	32.5 (3.8)
% body fat	38.1 (5.7)
Waist Circumference (cm)	
Male	110.6 (9.6)
Female	104.8 (10.7)
Education (%)	
Less than high school	1%
High School	10%
Some College	21%
College Graduate	28%
Post-graduate Degree	40%
Medication Used (%)	
Antidepressants	12%
Antihypertensives	19%
Lipid-lowering agents	6%
Thyroid medications	9%

Data are presented as mean (± SD).

The clinical program began with 190 individuals. After 6 months, 144 individuals (76%) were still involved. A series of independent sample t-tests found no significant differences (P > 0.05) at baseline for individuals who dropped out of the program compared to those who completed the program on any of the demographic or study variables. At 24 months, 104 individuals (55%) returned for all or a portion of the follow-up testing. Individuals who returned for the testing did not significantly differ from those individuals who did not return on weight, BMI, HRQL, fitness level at baseline, or in percentage of weight loss experienced during the clinical phase of the program. The only significant difference between the two groups was that individuals who returned for testing at 24 months were slightly older (51.3 yrs) than those who did not return for testing (47.8 yrs., p < 0.05).

### Baseline Health-Related Quality of Life

Table 2 includes the baseline values for HRQL in the study sample as well as the age-specific population norms taken from the interpretation guide for the SF-36 [20]. One-sample t-tests indicated that at baseline, study participants reported greater bodily pain and lower vitality and mental health scores than age-specific population norms (p < .05). All of the other subscales did not differ from age-specific population norms.

**Table 2:** Health-related quality of life at baseline in the study and in the US population ages 45–54.

**Scale**	**Baseline**	**Age specific Population Norm**^**†**^
Physical Functioning	83.3 (14.6)	84.61 (21.1)
Role Physical	81.0 (30.6)	82.65 (33.1)
Bodily Pain*	66.0 (17.8)	73.12 (24.0)
General Health	74.7 (16.6)	71.76 (19.4)
Vitality*	55.3 (15.0)	61.79 (20.9)
Social Functioning	89.0 (16.6)	84.07 (21.8)
Role Emotional	85.3 (27.8)	83.60 (31.4)
Mental Health*	70.8 (12.4)	75.33 (17.9)

Physical Composite Score	48.9 (7.05)	49.37 (10.4)
Mental Composite Score	49.9 (7.62)	50.32 (10.3)

Note. ^†^Given the demographics of the study sample, we used the combined scores for men and women ages 45–54 as the age-specific population norm.

* indicates a significant difference at baseline from the age-specific population norm for the scale.

### Changes in Health-Related Quality of Life

Mean weight loss at 6 months was 5.6 kg (6.1%) following the 6 month clinical intervention and 3.4 kg (3.7%) and 2.7 kg (3%) at the 12 month and 24 month follow-ups. Thirty percent of the sample that returned for testing had maintained a weight loss of at least 5% at 24 months. Repeated measures MANOVAs (n = 91) were used to examine changes in HRQL for the SF-36 subscales and physical (PCS) and mental (MCS) summary scales from baseline to the follow-up at 2 years with extended care group assignment and weight loss group as between subjects factors.

**Figure 1:**
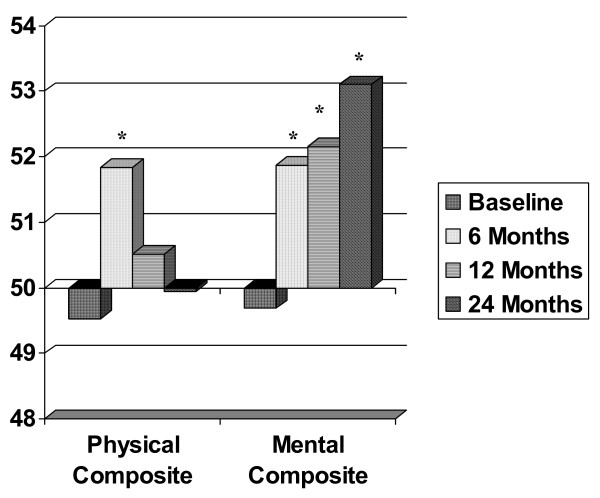
**Changes in the MOS SF-36 Physical and Mental Composite Scores**. Note. * indicates that the value was significantly different than at baseline (p < 0.05) using Bonferroni corrections from the repeated measures analysis.

The first analysis examined changes in the composite scales and indicated a significant multivariate Time main effect (F (6, 85) = 4.33, p < .001). There were no significant main effects or interactions for weight loss group and extended care group assignment (p > 0.05). There were significant univariate Time effects for both MCS and PCS (p < .05). Post hoc t-tests using Bonferrroni corrections indicated both MCS and PCS increased post intervention. However, PCS had returned to baseline levels by 1 year, but MCS remained higher than baseline at both 1 and 2 years. Figure 1 presents the changes in the composite scales from baseline to the 24 month follow-up.

**Figure 2:**
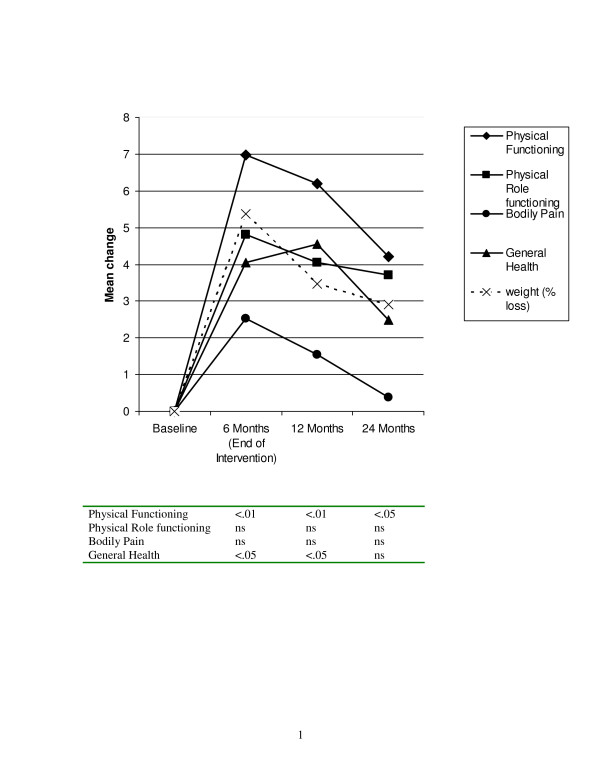
**Changes in physical health-related quality of life (MOS SF-36)**. Note. P-values in the table are for each time point compared to baseline using Bonferroni corrections from the repeated measures MANOVA.

**Figure 3:**
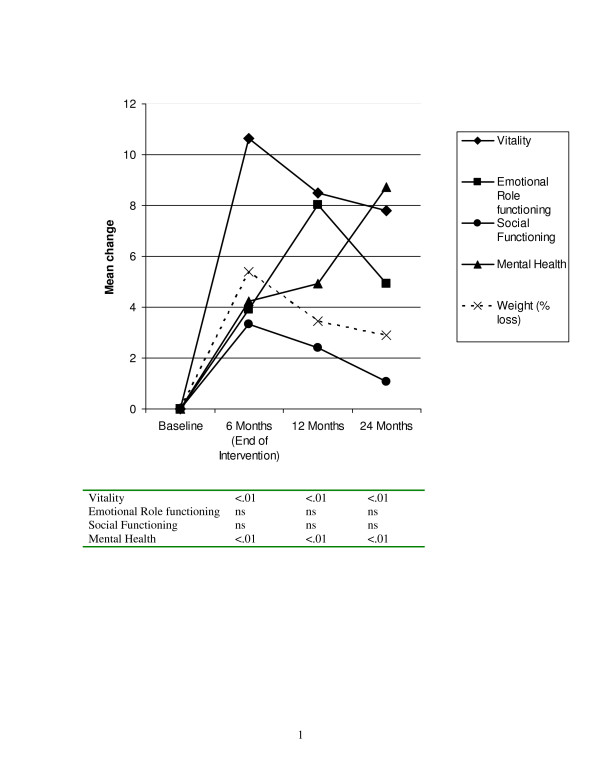
**Changes in mental health-related quality of life (MOS SF-36)**. Note. P-values in the table are for each time point compared to baseline using Bonferroni corrections from the repeated measures MANOVA.

A separate analysis was conducted on the SF-36 subscales was conducted to determine specific factors that were driving changes in the composite scale, this analysis again found a multivariate Time main effect (F (24, 67) = 4.36, p < .001). There were no extended care or weight loss group main effects or interactions. Univariate analyses indicated that significant time effects (p < .01) for the physical functioning, general health, vitality, and mental health subscales. Figure 2 presents the changes in physical health related quality of life plotted with change in weight. There were significant increases in both physical functioning and general health by the end of the intervention. Physical functioning remained higher than baseline at the 24 month follow-up, but general health was not statistically different than baseline levels by 24 months. Figure 3 presents the changes in mental health-related quality of life. Both vitality and mental health improved by the end of the intervention and remained at greater than baseline levels at the 24 month follow-up. When examining the patterns of change in relationship to percent weight loss, it appears as if the physical components of HRQL more closely paralleled weight loss, whereas the mental components of HRQL, especially mental health, did not necessarily track the weight loss pattern.

## Discussion

The current study provides further evidence that behavioral intervention in combination with diet and exercise produces modest long term weight loss maintenance and improvements in physical and mental quality of life measures. Study participants completed a 6 month behavioral intervention focused on increasing physical activity and adopting a healthy diet. At the end of the 6 months, the participants were randomized into two extended care treatment arms that received mailed intervention materials. At the end of two years, the participants maintained a 3 kg weight loss and 30% of the sample that returned for testing retained at least a 5% weight loss.

Many studies have shown that increasing levels of overweight and obesity are associated with decrements in the HRQL [12,22]. Although other studies have found decrements in HRQL across all of the subscales, the current study sample, although overweight and obese was only below the age-specific population norms in bodily pain, vitality, and mental health, and therefore may not be obese enough to have impairments across all aspects of HRQL. Kolotkin and Crosby's [22] examined HRQL by BMI level and did not find consistent differences until individuals had BMI's greater than 35 kg/m^2^. The mean BMI in the current study was 32 kg/m^2^. One exception has been physical functioning, which has been shown to be impaired at BMI levels greater than 27–30 kg/m^2 ^(e.g. [23-25]). However, that finding was not replicated in the current study.

Both the mental and physical composite scores improved at the end of the 6 month intervention and this was driven by changes in the physical functioning, general health, vitality, and mental health subscales. This parallels the findings of many other studies that have examined modest weight loss. For example, Fontaine et al. [26] studied 38 adults in a 13-week weight loss treatment program. Study participants lost an average of 8.6 kg and they reported improvements in physical functioning, role-physical, general health, vitality, and mental health. In a 12-week study, Rippe and colleagues [27] reported improvements in physical functioning, role physical, and mental health in 30 participants that lost 6.1 kg. A prospective analysis of the Nurses Health Study [28] reported that women that lost weight improved their physical functioning vitality, and bodily pain. In a study of a 4-month very low energy dietary intervention, there were transient improvements in many of the SF-36 scales [14].

Taken together these results suggest that it is possible to improve health related quality of life using behavioral interventions. Previous studies have consistently found improvements in physical functioning and many have found improvements in mental health, vitality, and role physical. The current study supported the improvements in physical functioning and also found support for improving general health, vitality, and mental health at the end of the 6 month intervention in which there was a moderate weight loss.

Given the ability of weight loss interventions to improve HRQL, it is necessary to examine long terms changes and what happens after the weight loss intervention ends. In the current study, at 1 year the scores on the physical composite scale were not significantly different than baseline levels, however the mental composite scale and physical functioning, general health, vitality, and mental health subscales all remained above baseline levels. At the 24 month follow-up, participants retained their improvements above baseline in the mental composite scale and the physical functioning, mental health, and vitality subscales.

The results of the current study have many similarities to the only other 2 year follow-up study of which we are aware [15]. Kaukua et al. [15] reported modest weight loss at 2 year follow up with 1/3 of patients maintaining ≥ 5% weight loss. In the current study, 30% of the study sample maintained a 5% weight loss at 24 months. There was a peak of improvements for many of the HRQL measures at the end of the 6 month intervention, followed by a gradual return towards baseline which mirrored the changes in weight. Kaukua et al. [15] reported a similar pattern, but only physical functioning remained improved over baseline levels at 2 years. The mental health subscale was the only exception, in that it increased over the entire 24 months of the study. Unfortunately we do not have data on changes in anti-depressant medications or enrollment in psychotherapy that might help explain this pattern.

In contrast to our study, Kaukua et al. [15] reported significant group differences when examining weight loss categories. In particular, they found that a 10% weight loss was necessary for improvements in physical functioning, physical role functioning, bodily pain, general health, vitality, and mental health. The results must be interpreted with some caution, as there were only 9 participants out of the 126 in the study that maintained a weight loss greater than 10% of their initial body weight. The current study used a cutoff of 5% weight loss or greater (30% of participants) and found no main or interaction effects. The lack of significance of the amount of weight loss on changes in HRQL has been previously reported. Kolotkin et al. [5] reported that only 14% of the changes in HRQL scales could be explained by weight loss. Similarly, Mathias et al. [29] reported that only 2 of 7 quality of life measures were different among individuals who lost greater than 5% of their weight compared to those that had stable weights (± 5%) and those that gained weight (> 5%). Fontaine et al. [16] also reported no difference among weight loss maintainers or regainers.

There is clearly a need to develop a better understanding of what is leading to improvements in HRQL among overweight and obese adults beyond weight loss. It is possible that behavioral factors such as exercising and changing diet can explain the improvements in HRQL [11]. It is also possible that the social interaction and support of the weight loss intervention is responsible for some of the improvements in HRQL. There is also a need to understand how to maintain improvements after completion of the intervention. In the current study, despite participants regaining weight, there were still improvements in vitality, physical functioning, and mental health at 24 months. An understanding of what programmatic aspects influence HRQL may help in the development of interventions that can foster continued improvements even after the formal intervention is over.

The majority of studies on obesity and HRQL have been examined from the perspective of surgical and/or pharmacological treatment for the severely obese. This study adds to the growing literature on the effects of behavioral interventions in producing more modest changes in weight that also can positively impact participants' quality of life. Further research is needed to examine the differential effects of very low energy diets, low fat diets, and low-carbohydrate diets. As research begins to suggest that the different diets may result in similar long-term weight loss results [30], it is possible that there may be differential effects on quality of life that are impacted by participants feelings of food choice and caloric restriction. It is also possible that different exercise prescriptions, such as different intensities and formats, may have differential impacts upon HRQL outcomes.

In general, the results of the current study are consistent with the few existing long term studies on health related quality of life and weight loss. However, there are several limitations to the current study. The current study only used a generic measure of HRQL (SF-36). Our results may not be the same if we used the obesity specific scale, such as the Impact of Weight on Quality of Life scale. The study should be replicated using multiple measures, including obesity specific and general HRQL. The current study sample was primarily white, female, and well-educated. A limitation of our analyses was the need to have complete data across all four time points. Therefore we were only able to analyze 48% of the participants that were originally randomized into the trial. The individuals who participated in the study were volunteers; therefore, they may differ from general population on some important characteristics. There is some research to suggest that individuals who seek out clinical treatment for obesity are more likely to have HRQL impairments than those not seeking to lose weight [31], although the current sample was relatively similar to age norms for HRQL. Therefore, replication should be done using different samples to increase generalizability.

## Conclusion

In conclusion, individuals were able to achieve significant improvements in HRQL following a 6-month behavioral intervention and were able to maintain many of those improvements at a 24 month follow-up. However, improvements in HRQL did not appear to be dependent solely on weight loss. More study is necessary to determine the correlates of improvements in HRQL with behavioral programs aimed at producing moderate, sustainable weight loss.

## Competing interests

The author(s) declare that they have no competing interests.

## Authors' contributions

BB drafted the manuscript and conceived of and conducted the analyses. DR conceived of the study, participated in its design and implementation of the exercise intervention, and helped to draft the manuscript. GD helped conduct analyses and draft the manuscript. LR designed and implemented the behavioral intervention. GG designed and oversaw the nutritional components of the intervention. MC helped conceive of the project and provided input in drafting the manuscript.
